# Ruptures as Imagined and Theorized: Symbolic Resources for Dealing With the Unexpected

**DOI:** 10.1007/s12124-025-09966-9

**Published:** 2025-12-10

**Authors:** Alex Gillespie, Tania Zittoun

**Affiliations:** 1 Department of Psychological and Behavioural Science, London School of Economics, London, United Kingdom; 2 Department of Psychology, Oslo New University, Oslo, Norway; 3 Institut de Psychologie et Education, University of Neuchatel, Neuchatel, Switzerland

**Keywords:** Theory, Narrative, Crisis, Rupture, Symbolic resources

## Abstract

What is the relationship between our theories of rupture (which view it as a basis for learning and development) and narratives of rupture (myths, novels, films, etc.)? On the one hand there are striking convergences. Both entail a variant of the following sequence: steady state, breach, crisis, and resolution or transformation. But, on the other hand, there are important differences. Narratives are multi-functional: they allow us to approach our fears, be entertained, pre-imagine crises, and they can become symbolic resources within crises, helping us to navigate ruptures. We argue that, rather than being mirrors of rupture, narratives of rupture are help us to navigate ruptures. This is formalized in model that shows how narratives enable us to pre-imagine ruptures, identify the early warning signs of a rupture, support our problem-solving, and subsequently narrate our rupture experiences. The model emphasizes the limits of narratives as symbolic resources within ruptures. Narrative guidance can fail, canalizing and constraining semiotic mediation in unhelpful ways. Due to inherent limitations of narratives as resources for ruptures, we propose that narratives also often provide meta-cultural guidance to ignore cultural guidance.

## Introduction

It is widely assumed that disruptions stimulate learning and development. The idea is that ruptures reveal a disconnect between representations and immediate experience that motivates semantic work to repair the representations. Repeatedly, research has shown how personal and collective ruptures are the disorienting engines of human and societal development. The underlying assumption is that semiotic reconstruction is prompted by our aversion to ruptures.

However, the literature on narratives focuses on how we enjoy and even savor ruptures. Myths, stories, and films all contain ruptures. Children’s games and practical jokes create enjoyment out of imagined breaches. A story that follows expectation is boring. It is hard to think of a myth, novel, or film that does not have a destabilizing rupture in it. And it is impossible to tabulate how many ruptures we have vicariously experienced in narratives. The puzzle is why we savor ruptures in our imagination, when we avoid them in our daily life?

This tension raises several questions. How similar are imagined and actual ruptures? Are imagined ruptures attempts to master our fears? Or are they resources for navigating actual ruptures? How do narratives of rupture interact with actual ruptures? At which precise moments in an actual rupture do imagined ruptures intervene, in what ways, and with what consequences?

We review the literatures on theorizing and narrating ruptures. There are striking similarities, but, there are also important differences. Integrating both literatures, we propose a model of how narratives of rupture are woven throughout the experience of actual ruptures. Symbolic resources, such as narratives, are used to anticipate ruptures, recognize their early warning signs, navigate them, support transformation through them, and make them narratable in retrospect. In this way narratives of rupture are not descriptions of rupture, but active ingredients within ruptures. Moreover, we propose that where narratives fail, when they become unhelpful for navigating ruptures, the narratives themselves often remind us of the need to go beyond their own guidance, to ignore the cultural expectations, and take a step into the unknown.

## Reducing Surprise: Theories of Rupture

The future will always exceed our expectations. Events are always more abundant than our representations (Feyerabend, 2001). This gap has motivated reducing surprise throughout human history (Friston, 2010). We use calendars, weather forecasts, and predictive modelling to reduce future uncertainty. Moreover, we use technologies, such as homes, thermostats, and fridges to create cocoons of stability within an uncertain world.

Culture guides us into the future and shapes our expectations for the future (Valsiner, 1998). Culture is the sedimentation of countless past experiences, packaged into cultural resources, that help us navigate the future. Our culture provides expectations about what will happen tomorrow, at the dentist, and next winter. Culture guides us to plant in the spring, to bring an umbrella if rain is forecast, and be careful taking on too much debt.

Science also reduces surprise. Theories are compared with observations, and unexpected results are meant to drive revisions (Ziman, 2000). Bayesian statistics explicitly measures how surprising an observation is given ‘priors’ (e.g., expectations informed by theory and previous observations). Good theories open the world to interesting, meaningful, and predictable interventions; bad ones create uncertainty (Gillespie et al., 2024). Of course, the self-correcting nature of science is not perfect; some domains suppress anomalies (Vazire & Holcombe, 2022). But, overall, science aims to reduce future uncertainties.

Ruptures are both problems and opportunities. They expose the limits of our cultures, narratives, and theories – reminding us that the world is more abundant than our understanding. But, they also motivate us to repair, to stitch our expectations back together, domesticate uncertainty, and reweave our comforting cocoons of stability. Thus, ruptures simultaneously reveal a failing in cultural guidance and provide an opportunity to improve future cultural guidance.

### Rupture-elaboration Theories

Attempts to theorize the role of ruptures in learning and development are longstanding. We will review the recent history of these theories from Dewey onwards. While not all these theorists referred to each other, they were all grappling with the same issue: how ruptures lead to semiotic elaborations which in turn, can lead to transformations (personal or collective learning and development).

Dewey (1896) was among the first formalized to a rupture-elaboration model, which he termed the perception-action-consequence loop. He argued that when perception, action, and consequences are in aligned, when what is expected is what is experienced, then humans are experientially embedded and absorbed in their activity (Benson, 1993). However, when outcomes deviate from expectations (e.g., a child reaching for a flickering object expecting a shiny toy but experiencing a candle), the perception-action-consequence loop breaks down. Cognition, Dewey argued, arises naturalistically within such irritations. Thinking is the attempt to reconstruct perception and response so as to fuse expectation and experience back together again. “Life”, Dewey (1922, p. 179) wrote, “is interruptions and recoveries.” The interruptions stimulate thinking, and the recoveries are the reorganizations of the cognitive system to reduce uncertainty.

James (1907, pp. 59–60) extended Dewey’s influential model by introducing the idea that learning through ruptures often entails resistance:

“The process here is always the same. The individual has a stock of old opinions already, but he meets a new experience that puts them to a strain. Somebody contradicts them; or in a reflective moment he discovers that they contradict each other; or he hears of facts with which they are incompatible; or desires arise in him which they cease to satisfy. The result is an inward trouble to which his mind till then had been a stranger, and from which he seeks to escape by modifying his previous mass of opinions. He saves as much of it as he can, for in this matter of belief we are all extreme conservatives. So he tries to change first this opinion, and then that (for they resist change very variously), until at last some new idea comes up which he can graft upon the ancient stock with a minimum of disturbance of the latter.”

James’ observation is that people don’t like to revise their beliefs – even when confronted with robust experiences. People look for the minimum viable change. His ideas foreshadow the idea that there can be defensiveness on the threshold of learning (Gillespie, 2020), and also a broadly Bayesian view of belief updating based on evidence (Hartigan, 2012). However, we now also know that people often don’t experience much “inward trouble” from contradicting beliefs (Panagiotou & Kadianaki, 2019).

Piaget (1936, 1977) also used a variant of the rupture-elaboration model, and he introduced an important innovation. He conceptualized learning in terms of cognitive systems seeking equilibria with experience. However, if experience conflicted with expectation, then there would be disequilibrium. Equilibrium, her proposed, could be re-established in two ways. The disruptive experience could be *assimilated* using pre-existing schema. This approach essentially avoids the opportunity to learn; the new experience is forced into the existing templates. Examples include the child which ignores evidence for the conservation of liquid, or scientists who assimilate an anomaly into an existing theory. *Accommodation*, on the other hand, entails adjusting the cognitive schema to make the novel experience intelligible. Accommodation is learning, because the cognitive system adjusts to the experience. Examples include the child which transitions to learning the conservation of liquid, or scientists developing a new theory or paradigm to explain an anomaly. Accommodation is any modification of the assimilatory schema, or cognitive system, brought about by new experiences (Block, 1982).

Bateson (1972) also used a rupture-elaboration model to theorize learning at a more abstract level. His influential distinction between single-loop and double-loop learning is similar to Piaget’s distinction between assimilation and accommodation. Single-loop learning occurs when there is adjustment of action toward a goal without questioning the goal or any underlying assumptions. For example, asking someone the time and being told it is 2pm might result in learning the time without any deeper learning (e.g., underlying goals and assumptions about time are unchanged). Double-loop learning entails changing the context of the single-loop learning. Double-loop learning is when people modify their goal, belief, value, or assumptions. For example, learning that because it is 2pm one is late for a meeting, might prompt deeper learning about one’s self-image as punctual. Again, as with Piaget, it is contradictions between expectations (I’m on time) and experiences (I’m late) that pushes learning.

Working at the level of collective knowledge construction in science, Kuhn (1962) observed rupture-elaboration sequences that included something similar to double-loop learning.

Science seeks observations that challenge, or rupture, its beliefs or theories. Kuhn observed that scientific progress was not linear, but, went through paradigm shifts. First, there is normal science, with puzzles being solved within the paradigm, fleshing it out. Second, there are anomalies, that conflict with the dominant paradigm, but which are often ignored or even suppressed. Third, as anomalies accumulate, there is a crisis, and alternative paradigms are explored. Fourth, a new paradigm emerges that can explain the anomalies, but which is incompatible with the previous paradigm. This new paradigm becomes the new normal science – until the next scientific revolution. The key idea that Kuhn introduced is that the development of scientific knowledge is not linear, there are discontinuities. In the terminology of Bateson: the leap from the old paradigm to the new entails double-loop learning that overturns taken-for-granted assumptions.

Another tradition, working at the collective level is social representations theory. Inspired, in part, by Piaget, Moscovici (2008) was trying to understand, at a collective level, how novelty was incorporated into commonsense. He investigated how psychoanalysis, as a new idea, was anchored in and objectified through pre-existing ideas and metaphors. The focus of social representations is on ‘making the unfamiliar familiar’, and it has been applied to nuclear power, genetic modification, and artificial intelligence (Bauer, 2014). The focus of this tradition is more on assimilation than accommodation. But, it powerfully reveals the role of pre-existing culture in ‘domesticating’ novelty, making it communicable and actionable. This tradition, however, has increasingly considered the microgenetic processes through which social representations change, for example through communication, or even the intra-psychological vacillation of ideas.

Yet another approach to rupture-elaboration sequences, especially in organizations, is sensemaking (Turner et al., 2023). Although sensemaking can be an individual process, the focus is usually on group dynamics. For example, an airplane crew dealing with a bird-strike or failing instruments, or a medical team making sense of statistics exposing high mortality rates (Weick & Sutcliffe, 2003). There are usually some team members speaking up about the problem, and there are others either listening or not (Pandolfo et al., 2025). Weick (2010) added the idea of ‘enactment’, namely, that sensemaking often entails dynamic transactions with the environment. Thus, sensemaking is not merely semiotic (psychological) or verbal (communicative), it is also practical. People try this or that. The crew reboot the instruments, use manual over-ride, or re-position the plane. These actions are part of the sensemaking, because the consequences of the actions feedback into the understanding of what is going on. While the idea of enactment is useful, the sensemaking tradition rarely analyses the micro-semiotic processes of sensemaking.

Valsiner’s (2003) theory of enablement adds an in-depth analysis of the semiotic processes within a rupture. He situates semiotic mediation within a basic rupture-elaboration framework: humans are oriented towards the future, but, en route, they encounter obstacles (either from the world, or from contradictory impulses) that trigger semiotic activity to enable their journey to continue. Signs enable distanciation from the rupture (i.e., being able to talk about it), and imagining possible routes through the rupture (e.g., practical or emotional guidance). The key idea is that signs are semiotic tools that guide and constrain thought and action, creating channeled possibilities, within the uncertainty of a rupture. Valsiner demonstrates how, within the moment of a rupture, signs layer up, each new sign reacting to the prior sign, creating semiotic chains that can open and close opportunities within a rupture.

Zittoun and colleagues (Zittoun, 2006;2003) introduced the idea of using symbolic resources within ruptures. In contrast to material resources (e.g., money, techniques for preventing the spread of a virus) and social resources (e.g., access to advice, support, legitimacy), symbolic resources help people to think through a rupture. A symbolic resource is a bounded cultural element (such as a book, film, prayer, or ritual) that is constituted by being drawn into the rupture and used semiotically within the rupture. The focus is on particular social, cultural and temporal contexts (i.e., specific ruptures) and the learning or transition that results from the rupture. Building on Valsiner’s (2003) focus on semiotic mediation, this approach has examined the micro ways that different individuals have navigated ruptures using symbolic resources, and the specific ways that they engaged with symbolic resources in diverse ways (e.g., problem solving, containing emotion, imagining possible futures).

Since Dewey’s initial formalization the rupture-elaboration model has been greatly refined. Dewey and James developed the model through armchair theorizing. Subsequent researchers advanced the model through empirical research. Piaget, Kuhn, and Bateson, each in different domains, introduced the idea that for ruptures to lead to development, they must entail a reorganization of the existing schemas or paradigm. Weick revealed the role of sensemaking activities, whereby the consequences of actions feedback into understandings of the rupture. Valsiner and Zittoun and colleagues have revealed the micro-semiotic processes through which people think-through ruptures; how semiotic mediators and symbolic resources are utilized in the moment to distanciate from the rupture, generate plausible paths of action, and prompt reflexivity and creativity within the rupture.

All the rupture-elaboration conceptualizations distinguish between what we will call expectation and experience. Expectations are culturally-guided understandings of what should happen; sedimentations of personal and collective past experiences that lean into the future and domesticate uncertainty. Experience refers to in-the-moment perceptions, obstacles, thoughts, and actions. Although experiences are shaped by cultural guidance, they are the moment when reality, other people, or unfamiliar things disrupt expectations. Usually experience follows expectation, but, in a rupture, expectation lags experience. In this sense, ruptures are the ‘frontier’ of human individual and cultural development (Simao, 2003).

### When Cultural Guidance Fails

The role of culture in a rupture is twofold and contradictory. Culture is the sedimentation of the countless experiences of our ancestors. It is meant to make our world predictable, channel human thought and action, and provide guidance (Valsiner, 2014). It is a powerful toolbox for dealing with a myriad of uncertainties. But culture also narrows expectation, constrains thought, and channels semiotic mediation in potentially misdirected ways. Culture makes us see the new in terms of the old, and thus potentially obscures novel particularities and unfamiliar avenues for thought and action.

Consider the case of the Grenfell Tower fire in London in 2017, where 72 people died (Cornish, 2021). Each resident who called emergency services was given the same advice: to stay-put in their apartment. The building was designed so that fire would not spread between apartments. But, the tragic rupture was that the fire was spreading between apartments due to new cladding. Accordingly, the residents who ignored the safety plans and advice and evacuated were saved. Thus, there was not only the immediate problem of the blaze, but, there was a secondary problem that the cultural guidance (i.e., to stay-put) was wrong.

Or, consider the 1949 Mann Gulch disaster in the USA (Weick, 2009). Fifteen firefighters were parachuted into an ostensibly straightforward fire. However, due to unusual winds, the fire escalated, and began to surround them. Their expectations and their training blocked them from seeing the changing situation. When the team realized the problem, they experienced *vu jade*, the opposite of *déjà vu*. This is the feeling of never having seen it before, of a complete collapse of expectation. As their experience of the fire overtook them and their expectations, the team leader, Doge, told everyone to drop their tools and burn the path. They did not – lighting a fire when becoming surrounded by a fire made no sense. Doge lay down in his burnt clearing, letting the fire burn around him, and survived. Thirteen firefighters died trying to outrun the blaze. Doge survived by abandoning his expectations and training. Thus again, the cultural guidance (initial briefing, training with similar fires, how to escape a fire) failed.

These cases are not isolated. Famously, in 2009 Flight 1549 struck a flock of birds, lost power in both engines, and it was unclear if they had enough altitude to reach an airport. Captain Sully (portrayed by Tom Hanks in the film *Sully*) made the unprecedented decision to land on the Hudson River (Garcia, 2016). He defied protocols but everyone on board survived. Similarly, during the 2010 Deepwater Horizon oil rig explosion in the Gulf of Mexico, which claimed 11 lives, both primary and secondary muster stations were engulfed in flames. The survivors were those who abandoned protocol and leapt into the ocean (Reader & O’Connor, 2014).

Abandoning cultural guidance is often integral to resolving crises because crises occur, almost by definition, when plans and expectations fail. The crisis was not meant to happen – otherwise it would have been averted. Of course, we often have plans for what to do in a crisis (stay put, land at the nearest airport, gather at the muster station), but, a crisis always has the potential to break beyond these protocols. Blindly following expectations (plans, protocols, cultural guidance) for what to do in a crisis risks failing to see the crisis for what it is, namely, a unique event that may (or may not) be comparable to prior crises.

Cultural guidance enables us to navigate daily life with surprisingly little thinking. But, sometimes, it fails. Sometimes we are exposed, with few cultural supports. Sometimes we need to stop and think (Arendt, 1978). In these moments of breakdown, double-loop learning is required. We need to reach deep into culture, go beyond the expectations for the situation, and construct new and often heretical paths of action (e.g., burning grass within a fire, abandoning the muster stations, rejecting the stay put policy). But how do we do this? How can we step outside the canalized paths that culture has set for us? In short: what is the cultural guidance for when cultural guidance fails?

## Savoring Surprise: Imagining Ruptures

Although we try to avoid ruptures in our daily lives, we savor them in our imagination. Infants, once they have established basic routines, are especially interested in surprises and disrupting routines, whether in interactions, in the movements of objects, or in patterns of activity (Trevarthen, 2015). Their increased interest in surprises is marked by increased attention to laughter, excitement, shrugs, and questions (Butler et al., 2020). The pleasure they get from these deviations is visible in their early joy in peek-a-boo games or early clowning (Mireault & Reddy, 2016). In good enough environments, surprise can be cultivated into curiosity, which is a deliberate attention to rupture, and the motor of learning (Engel, 2015; Zittoun, 2023).

Adults also enjoy playing with ruptures. Practical jokes are well crafted ruptures in expectation (creating a scare, misperception, or foolish act). Many TV shows, such as *Candid Camera*, have entertained millions by rupturing people’s expectations for the pleasure of the audience. Verbal jokes also entail a jolt in expectation – without this shift of perspective, there could be no humor (Dunbar et al., 2016). Moreover, ruptures are central to narratives – from myths to blockbuster movies.

### Ruptures Pervade Narratives

Narratives are an essential and long-standing feature of human thought. Bruner (1986, 1990) argued that, in addition to more formal cognition that tries to predict and control, the human mind also tries to explain and understand through narratives. Narratives, he proposed, give coherence and meaning to experiences and enable us to operate in ‘possible worlds’ – which allow humans to rehearse, simulate, and explore imagined alternatives.

Intriguingly, narratives have a similar structure to the rupture-elaboration theories. Consider myths. Campbell (1949) analyzed hundreds of myths from diverse cultures and argued that there was an underlying ‘monomyth’. This core template myth, he argued, was ‘the hero’s journey’ and it had 17 stages that he grouped into three phases: (1) departure, where the hero leaves their routine life and they cross a threshold into the unknown; (2) trials, misconceived approaches, allies, resources, and struggle all leading to success, and; (3) returning to the previous daily life, but as a transformed person, or in a transformed world.

Campbell’s (1949) monomyth has become self-reinforcing. It is used as a template for people’s self understanding (Rogers et al., 2023) and it is explicitly utilized in many blockbuster narratives (Vogler, 2017). One can find traces of it in books such as Harry Potter (who discovers he is a wizard, leaves his mundane life, endures trials, triumphs, and returns) and in films like the Matrix (where Neo discovers the matrix, leaves his mundane life, endures trials and saves humanity).

However, the idea of the monomyth has also received criticism. It is overly simplistic to squeeze the entire diversity of human myths into a 17 linear stages; it quashes subtleties, nuances, and changes over time. There are variations between cultures (Wertsch, 2008) and also within cultures. Indeed, systematic analyses of thousands of narratives find multiple story arcs (Boyd et al., 2020; Reagan et al., 2016). Emotional and plot arcs move in multiple ways that cannot be reduced to a monomyth. Nonetheless, although Campbell’s 17 stages might be too constraining, all these story arcs do have moments of rupture and narrative tension.

Support for the idea of a simpler rupture-elaboration template underlying many narratives goes back to Aristotle. Aristotle’s (2011) *Poetics* argues that good narratives should have peripeteia (a reversal) and anagnorisis (realization). Peripeteia is an unexpected reversal of fortune stemming from the action of the protagonist. This sets up anagnorisis, which is a moment of realization, where the truth of the situation is revealed to the protagonist. Consider the famous short story: ‘For sale. Baby shoes. Never worn.’ If the third sentence was ‘Yellow laces’ there would be no rupture, no anagnorisis, and arguably, no story. This simpler narrative template is more generalizable than Campbell’s monomyth.

Bruner (1986, p. 21), when discussing the possibility for a “timeless underlying theme” to narratives is also suspicious of any over-specified template. Yet, Bruner argues, there is undeniable commonality to narratives: they contain a plight into which characters have fallen – a rupture. At a most general level, Bruner (1986) argues, the structure typically (but not always) comprises: steady state, breach, crisis, redress. Without a breach of the canonical state, stories are boring. Stories of the steady state are not interesting stories. Narratives rarely recount our mundane lives, instead, they seem to home in on ruptures. “Narratives”, Bruner (1986, p. 16) writes, “start with a canonical or ‘legitimate’ steady state, which is breached, resulting in a crisis, which is terminated by a redress”. While we agree with Bruner’s broad outline for a core schema, we take issue with the term ‘redress’, as we place more emphasis on ‘transformation’ – of the world or the protagonist.

### Why Are Ruptures so Prevalent in narratives?

Several authors have tried to explain why narratives of rupture are so widespread across times and cultures. The explanations have ranged from psychoanalytic, through cognitive, to more practical.

Since Freud (1899, 1907) it has been common to understand dreams and narratives as a way to experience fantasies without endangering our daily life. In effect, for Freud children have the license to explore their desires through pretend play; at adolescence, when people have to conform to the social order, they lean to internalize or repress their fantasies, wishes and desires. Artists, for example, can give shape to these socially unacceptable wishes and desires in narratives or other artwork. Reading novels, or having cultural experiences, enables us to vicariously experience our own fantasies, wishes and desires in a culturally sanctioned way. When we experience narratives (plays, films, myths) we know they are ‘not real’, but, the emotional impact can be real. Because these are transposed into an artistic form, they can become what Freud would call “sublimated”, that is, elaborated through semiotic processes and detached from the urge to be satisfied in reality.

Freud (1899), Winnicott (1971), Vygotsky (1972) and many others have demonstrated that art and fiction enable us both to contain and transform our affects in the safe space of imagination. In some cases, this can enable the fulfillment of wishes. From this standpoint, narratives of rupture might help us to overcome our fears (i.e., about various ruptures) and also satisfy deeper urges to be heroic, to master the world, and potentially ourselves.

Another explanation for why narratives of rupture are widespread comes from cognitive psychology. The idea is that our cognitive system has a predilection for certain symbolic forms, that we seek patterns, and like closure. Accordingly, our cognitive systems have selected stories that have rupture and resolution.

For example, studies of people reading narratives have found that ruptures in expectation, indicated by surprise, cause orienting responses – such as elevated pulse, pupil dilation, and increased galvanic skin response (Sukalla et al., 2016). These responses are independent of the prior level of engagement and suggest that narratives that exploit our attention to surprise will circulate widely. In a similar vein, Tobin (2018) argues that we expect the world to be predictable and orderly. Thus, plot twists, reversals, or ruptures violate these assumptions – thus grabbing our attention. Tobin argues there is pleasure in having our expectations upset and then reset. We enjoy the clarity of thought that follows a plot twist. Again, the idea is that rupture narratives have adapted to our cognitive structure.

A third interpretation is that narratives have a more pragmatic function. Campbell (1949) argued that myths are resources for dealing with life; for navigating death, loss, suffering, and fear; for living an authentic life; for overcoming challenges; and to encourage people to follow their dreams. Bruner (1986, 1990) argues that narratives help people make sense of ruptures and imagine alternatives within ruptures. They can support the pre-imagining of the rupture, preparing us for it, and within the rupture, they can support us imagining alternatives, and possible ways forward. In short, the rupture narratives are widespread not merely because they appeal to our cognitive structure, but because they are useful in navigating ruptures.

It is plausible that narratives serve all three broad functions: that they satisfy our wishes, appeal to our cognitive architecture, and act as symbolic resources within ruptures. However, this would greatly complicate their role as resources to help us navigate ruptures. If narratives have been created to fulfill heroic deeds, dramatic plot twists, profound transformations, and happy endings, then, how useful will they be within a real rupture? When navigating a rupture, what is needed is, perhaps, something less heroic, more pedestrian, and less dramatic; namely, something useful. The problem is that narratives that are successful outside of a rupture (giving expression to sublimated desires and appealing to cognitive processes) might not be useful within a rupture.

## Convergences & Divergences

There is a striking deep structural similarity between the rupture-elaboration theories of human transformation and the structure of many (but not all) narratives. The rupture-elaboration theories coalesce around the following sequence: taken-for-granted life, breach of expectation, semiotic elaboration, and transformation. Narratives tend to coalesce around the following sequence: steady state, breach, crisis, and redress. While we can debate the choice of terms and the extent of universality, the underlying similarity is striking. There are two plausible interpretations.

Maybe this similarity is evidence for the rupture-elaboration thesis. This convergence of theories of transformation (developed recently using empirical methods) and narratives (developed over thousands of years of human story-telling) onto a similar structure provides converging evidence that ruptures really are central to human development. It suggests that the rupture-elaboration sequence is, perhaps, the most fundamental and timeless engine of human development.

A more skeptical interpretation is that the rupture-elaboration theories have been seduced by the narratives. Like the Hollywood films that have reproduced Campbell’s monomyth, maybe our theories are actually derivative of, or formalized descendants of, the narratives. The rupture-elaboration models began with armchair theorizing (Dewey, James), and, although they have become increasingly empirical, maybe they have not fully escaped their origin in narrative?

In support of this more skeptical interpretation, there are important differences between actual and imagined ruptures that are often overlooked. While narrated ruptures have a clear beginning and end, this is often not the case with lived ruptures. While imagined ruptures often conclude with transformation, there are many lived ruptures that either have no ending or no transformation. For example, a crash that leaves a loved one in a vegetative state can be an interminable rupture without transformation (Zulato et al., 2023). While narrated ruptures tend towards coherence and tying up loose ends, actual ruptures often lack coherence and have plenty of loose ends. But, most importantly, in narratives there is the tendency for a happy ending, and in life, that cannot always be the case.

It is not obvious that personal ruptures such as becoming unemployed, ill, or losing a loved one will lead to transformation. How many times do people have revelations on New Years Eve, only to make the same New Years Resolutions the next year? Transformation is rare. Equally, there are many societal ruptures which, arguably, led to very little transformation. Public inquiries into societal failings routinely show the same underlying causes, without any subsequent systemic learning (Hald et al., 2025). Maybe the claim that ruptures lead to transformations is over-stated wish-fulfillment.

While transformations usually stem from ruptures, not all ruptures lead to transformations. Accordingly, we need a rupture-elaboration model that incorporates imagined transformations, but, does not assume that there will be actual transformations. Indeed, in addition to explaining how semiotic mediation within a rupture leads to transformations, we also need to understand how it does not; how transformations might be blocked.

## An Integrated Model

In this section we integrate the rupture-elaboration theories with literature on narratives to propose the anticipation-rupture-elaboration-transformation-absorption (ARETA) model. This model shows how narratives of rupture, as symbolic resources, can help us to anticipate, recognize, understand, and respond to a rupture. But, we also show how narratives can create obstacles within ruptures, channeling expectation in misdirected ways.

1. *Anticipation: pre-imagining*

Few ruptures truly come out of the blue, like a meteorite, in a world with no knowledge of meteorites. Most ruptures have been anticipated, or ‘pre-imagined’, through narratives. These narratives (myths, novels, films, games) allow us to vividly imagine numerous ruptures; to vicariously live diverse ruptures. This sensitizes us to the potential for ruptures: a taken-for-granted relationship could collapse, a visit to the doctor could uncover an illness, a routine flight could end in a crash.

Of course, some crises are more pre-imagined than others. Personal crises, such as illness, death, unemployment, financial ruin, and divorce have been widely explored in novels, plays, and films. More macro crises, such as wars and plane crashes are, arguably, even more thoroughly imagined, especially in blockbuster movies. Although it has not been a crisis yet, if AI does go rogue, it will be one of the most pre-imagined crises in history. Conversely, the 2020 pandemic had surprisingly little pre-imagination. One of the most genuinely surprising ruptures was the 9/11 terrorist attack on the World Trade Center in 2001. For most people, this was, until it happened, inconceivable – even among the intelligence communities (Syed, 2021). When ruptures are radically unexpected, they can so shatter expectation that they result in trauma (Theisen-Womersley, 2021).

Pre-imagination is not just through popular novels, films, and games. Organizations use a different range of symbolic resources to pre-imagine potential crises. The most obvious example is fire-drills, risk-assessments, stress-tests, scenario planning, and horizon scanning. But, they also conduct vivid simulations, for example, in surgery, aviation, and terrorist response. Military organizations run ‘war games’ to better anticipate dynamic and uncertain scenarios. Through these techniques, organizations pre-imagine crises – and specifically ensure that employees are familiar with the routines of crisis management.

The role of symbolic resources before a rupture, to anticipate potential ruptures, has been neglected in the rupture-elaboration models reviewed above. Instead, they have focused on the use of resources within ruptures. But, the role of symbolic resources begins before the rupture. The experiences of countless prior ruptures are packaged, and made available for vivid rehearsals. Thus, most people have encountered many more crises in imagination than in reality. Pilots might only encounter one crisis in their career – but they will have been in a flight simulator enacting crisis responses countless times. Equally, through symbolic resources, we have all pre-imagined countless crises that, hopefully, we will never live through (trauma, incarceration, wars, societal breakdown, climate collapse). Due to these symbolic resources, ruptures are, somewhat paradoxically, never true ruptures.

The extent of pre-imagining crises can also become problematic. Our technologies of the imagination have become increasingly powerful (computer generated images, virtual reality). More than ever before we can vividly, and emotionally, pre-imagining crises (from crashes to avalanches, from nuclear fallout to wars). This creates the potential for increased anxiety about potential crises (Beck, 1992). While some sensitization to ruptures is likely valuable, it is also possible that too much pre-imagination is incapacitating.

2) *Rupture: early warnings*

A rupture begins with no rupture. The transition into a rupture can be rapid or slow. For example, the time between taken-for-granted and this-is-a-crisis is very short in a car crash. Although, even in car crashes the transition is rarely instantaneous, and people are often too hesitant, suggesting that they are alerted to the danger, but not fully acting on it (Loeb et al., 2015). A slow transition, on the other hand, can last for weeks or years. For example, an unhealthy lifestyle leading to illness or a slowly unfolding climate crisis. In these latter cases, the lack of unambiguous early warning signs creates even more possibility for hesitancy.

Warning signs are ignorable signals that expectation and experience are out of alignment. Warning signs can come from the world: there is a twinge of foot pain, the dark clouds on the horizon, the unidentified caller asking for login details. They can come from other people: telling us that the clouds are ominous. They can also come from ourselves – in the internal dialogue that questions the pain, clouds, or the call - comparing them to prior experiences, to films, or to what we learn from reading into the issue.

A key feature of the warning signs is that they are not definitive – they can plausibly be ignored. This inherent ambiguity of early warning signs allows for multiple potentially incompatible interpretations (Abbey & Valsiner, 2005). For example, in the Bhopal tragedy, which was the most lethal industrial disaster in human history, early warning signs were ambiguous and thus ignored; one employee raised a concern about a potential leak, only to be told by a supervisor that the smell was mosquito spray (Weick, 2010). Or consider the case of someone experiencing shortness of breath; is it really worse than yesterday? Maybe it is normal? The ambiguity provides a space for self-deception.

The inherent ambiguity of early warning signs also allows for defensiveness, where people use a range of tactics to ignore early warning signs (Gillespie, 2020). This can happen when people avoid the problem (e.g., signs of illness, pandemic, or climate change), delegitimize it (e.g., ‘project fear’, ‘plandemic’, ‘climate alarmism’), or limit it (‘it is not that bad’, ‘we have moved on’). In the Bristol Royal Infirmary in the UK, where infants were dying unnecessarily during heart surgery, the clinicians dismissed the statistics because they claimed they had the most complex cases (Weick & Sutcliffe, 2003). In all these cases, defensive tactics are used to block semiotic escalation towards definition of the situation as a crisis.

Ruptures have varying degrees of actionability. Sometimes corrective action based on the early warning signs can avert the crisis (going to the doctor early, robustly investigating a concern). At other times, the options for corrective action are minimal (a meteorite, a tsunami). However, it is invariably better to recognize the early warning signs to enable either corrective action or preparation for the worst. And, in this regard, symbolic resources can help.

Narratives of rupture frequently include early warning signs, thus potentially sensitizing people to them. In Hansel and Gretel the house made of sweets is a too-good-to-be-true warning sign. In Little Red Riding Hood the voice of the disguised wolf is an early indication of the danger. Equally, in films such as *The Birds*,* Jaws*, and *Contagion* there are people, and especially the media, who downplay the emerging threat. Or, in the 1984 UK film *Threads*, which is about nuclear fallout, the opening is not focused on war, but it is constantly on TVs in the background, providing an ignorable sign of what is to come. Arguably, these narratives remind us that warning signs are often missed with catastrophic consequences. Arguably, if our collective imagination of ruptures never included warning signs, we would be even less attuned to them.

3) *Elaboration: canalization & constraints*

The elaboration stage begins with the acknowledging that there is a rupture. The past is gone; it is a pandemic, the fire is out of control. The rupture cannot be ignored. As old expectations crumble, disorientation sets in. Although the rupture may affect only one part of a person’s understanding, it can cast doubt on many others. Uncertainty can spread. Familiar footholds that once felt objective seem subjective. The situation, once clear and coherent, breaks apart into fragments of the old reality and scattered, often conflicting, hints of a new one. Semiotic elaboration asks: What is the problem? What caused it? What can be done? What is the way forward?

Addressing these questions entails both cognition and action. Action contributes to the sensemaking. On the one hand, ideas about the problem guide action (thinking it is a storm, leads to checking the weather forecast). But, on the other hand, the actions guide the ideas (e.g., trial-and-error experimentation to see what works to open the jammed door). Ideas guide actions which lead to consequences which guide ideas (i.e., enactment; Weick, 2010).

Addressing these questions can also be a social process. Children, when facing a challenge, ask adults for help. Adults, when facing big decisions, chat with trusted friends. And, in a crisis, people seek the advice of experts (doctors, solicitors, police). In organizational contexts, the ruptures usually occur within a team (flight deck, bridge, surgery team, police squad etc.). In these contexts, the sensemaking process is shaped not only by psychological processes, but also social process (Pandolfo et al., 2025). Do people speak up? Do some people dominate? Are people listening?

Crucially, for our analysis, these questions can also be addressed with cultural resources. Culture is not a monolithic set of expectations, guiding us on a single track. Culture is a bricolage, of overlapping, and often conflicting images, heuristics, metaphors, and ideas (Gillespie & Zittoun, 2010). Cultural resources refer to the entire stock of knowledge, rituals, practices, and norms that come from our individual and collective past (Swidler, 1986). Our focus is on symbolic resources, the subset of cultural resources, that pertain to bounded semiotic elements, especially narratives.

Symbolic resources get pulled into the sensemaking process, as aids to semiotic mediation (Zittoun et al., 2003). They contribute to the flash of ideas, the building of semiotic chains, and the grasping for ideas just beyond reach. The understanding of the rupture reverberates with associations from previous actual and imagined ruptures. The resources used and the semiotic chains constructed are heterogeneous. A person mourning a death might rail against the loss (‘my life is meaningless’) or take solace from religion (‘family reunited in heaven’). A person losing their job might blame themselves (‘I’m such an idiot’) or be inspired by movies to make a fresh start (‘this is my chance to pursue my dream’). A person struggling for a deadline might be tempted to give up (‘this is impossible’) or associate their effort with the hero’s journey, as just another trial (‘another challenge for me to conquer!’). Research has found that people who watched more horror movies were more resilient during the 2020 COVID pandemic (Scrivner et al., 2021).

Symbolic resources can be pulled into a rupture to speculate about potential causes, to generate possible actions, and to imagine possible futures (Bruner, 1986). Symbolic resources, as pre-created symbolic spaces, open up a space for imagination. Imagination weaves potentials and possibilities between the overlapping alternatives, and loosely connected scenarios and narratives (Zittoun & Gillespie, 2015). Without parallel narratives, and even implausible scenarios, the space for thinking would shrink.

Imagined ruptures can also infuse actual ruptures with hope; the possibility of heroic deeds leading to happy endings. Sometimes the rupture being faced has no end (internment without trial), entails an irreversible loss (of property or family), or is a terminal decline (illness, dementia). What if the rupture is unlikely to reward heroic deeds (armed robbery)? Maybe in these situations, even though they do not align with heroic narratives, the imagined ruptures might be a source of hope and resilience. Maybe the imagined ruptures keep alive the possibility for dramatic reversals, recoveries, and reunions.

However, imagined ruptures can also be barriers to dealing with the immediate rupture. The narratives come from the past, and have been crafted, in part, to serve our wishes (Freud, 1899). The rupture faced will always have novel aspects, and is unlikely to pander to our wishes. Accordingly, the narratives of rupture might obscure particularities or give false hope. Maybe the unfolding rupture will not follow the expected course, maybe it will not have a happy ending, maybe heroic deeds won’t save the day. In these ways, symbolic resources can lead to missed opportunities, unrealistic expectations, and a failure to learn. Thus, instead of learning from the rupture, it might be assimilated into a poorly fitting narrative template.

4) *Transformation: accommodation*,* double-loop learning*,* & paradigm shifts*

Transformation is rare – rarer than suggested by narratives and theories of rupture. Transformation occurs when the cognitive system needs to adjust to accommodate a new experience (Piaget, 1936), when the assumptions or goals become questioned with double-loop learning (Bateson, 1972), or when there is a paradigm shift in thinking (Kuhn, 1962). Without a substantial adjustment of the interpretative scheme in response to experience, there is no transformation.

Usually, the transformations are fundamental for the individual, but, not for the culture. For example, a child that realizes the conservation of liquid has had a cognitive revelation, but, it is not news to other people or the broader culture. However, sometimes the transformations are on the edge of culture, such as how to deal with an emerging and novel crisis (such as a modern pandemic, or Flight 1549). But, in the domain of science, the paradigm shifts caused by ruptures can be truly culture creating (e.g., the theory of relativity, the Copernican revolution).

Transformations imply that the internalized cultural guidance has failed. Almost by definition, if the cultural guidance was successful, then the rupture would not arise – or it would have been expected and prepared for. On the precipice of transformation the usual cultural supports are gone; it occurs at the edge of our own enculturation. Our cultural guidance has led us to a dead-end. So, what kind of cultural resources are available on the threshold of transformative learning or paradigm shifts? What resources might support a double-loop adaptation to a novel rupture?

A common theme in rupture narratives is that the protagonist has to leave behind the accepted wisdom, outgrow their mentor, and forge a new path in the world. Odysseus ignored advice, for example to avoid the Scylla and the Cyclops. His persistence led to suffering for his crew, but also rewards and his own transformation. The Buddha ignored advice not to leave his palace, but he did, and through the suffering he discovered, he became enlightened. Cordelia breaks with expectation by refusing to flatter her father, King Lear. By defying his command, she exposes the fatal flaw in his scheme to divide the kingdom.

This idea of resisting cultural guidance is even stronger in blockbuster movies. In *Star Wars New Hope* (1977) Luke Skywalker is meant to use a targeting computer to destroy an exhaust port on the Death Star. At the crucial moment he switches off the targeting computer and instead decides to ‘use the Force’. In *Top Gun* (1986) Tom Cruise is a pilot aptly called Maverik, who repeatedly brakes rules – this is superficially a weakness, but ultimately a strength. In the film’s climax he ignores orders to return, instead he re-engages the enemy, and saves his fellow pilots. In the *Harry Potter* movies, the young wizard repeatedly breaks rules – going into prohibited spaces, breaking curfews, and using forbidden spells. However, the rule-breaking, is in the pursuit of a higher goal – it is more attuned to the particularities of the challenge faced – and thus it enables his ultimate triumph.

Might these narratives of heroes breaking with expectation be cultural guidance reminding us to sometimes ignore cultural guidance? Potentially this is, paradoxically, a form of meta-cultural guidance for operating at the limits of cultural guidance. At the extreme we see it in Don Quixote, by Miguel de Cervantes. The elderly gentleman, having become seduced by chivalric romance narratives, becomes a hapless knight. Guided by these narratives he fights with friends, strangers, and windmills – only to be humiliated. Only on his deathbed does he realize the folly of chivalric fiction. Thus, like many more recent narratives, it includes a warning about relying too much on cultural guidance. Real heroes need to address the particularities of their own unique situation.

5) *Absorption: returning to culture*

Ruptures invariably end up as narratives, becoming absorbed back into culture. The breach, the back-and-forth elaboration, and the outcome are packaged into a sharable story. The rupture can be narrated as a turning point, as a lesson, as a resource for the future, as a warning tale, or as a funny story. If people share the story, it will spread, and become absorbed into culture and merge with the pre-existing narrative templates.

Ruptures are narrated through available cultural templates. Thus, again, the cultural narratives, previously used to pre-imagine and navigate the rupture, are now activated again to re-tell the rupture. In this process the specificity of the rupture is once again canalized by history.

Usually, ruptures are assimilated into the culture. In so far as the rupture is a familiar type, it will fit the established narrative and, in a Piagetian (1936) sense, become assimilated. If the rupture follows Bruner’s (1986) breach-crisis-redress schema, then there is no need to change the template, and the rupture will be classed as simply another instance of the standard type.

However, it is possible for cultural accommodation to occur. The narrated rupture might contain some novelty. This is especially visible in the domain of science. When a new theory or experimental result breaches expectation, it might require that the existing body of scientific knowledge accommodates it. This is what Kuhn (1962) called a paradigm shift.

## Discussion: Disentangling Imagined and Actual Ruptures

The rupture-elaboration theories, reviewed above, have been entangled with narratives of transformation. Both focus exclusively on the moment of rupture, and the potential within it. But, instead of mirroring rupture narratives, our theories of transformation through rupture need to rise above the narratives, separate from them, and incorporate them as active ingredients in the model.

The ARETA model expands the classic rupture-elaboration-transformation sequence (Bateson, 1972; Dewey, 1896; Piaget, 1936; Valsiner, 2003; Zittoun et al., 2003) by wrapping the start and end in narratives. Most ruptures are anticipated, or pre-imagined, and most ruptures are narrated after the fact. This model also differentiates the rupture-elaboration theories from narratives of rupture (Bruner, 1986, 1990; Campbell, 1949), because these first (anticipation) and last (absorption) stages of the ARETA model are rarely in narratives (i.e., a protagonist pre-imagining the forthcoming crisis, or narrating it afterward).

Additionally, to further disentangle imagined and actual ruptures, we need to be wary of the transformation bias. In imagined ruptures the hero often changes the world, bending it to their will. However, in actual ruptures it is often the world that changes the hero; they are bent to the will of the world. Ruptures can be more disciplining than liberating. Even societal ruptures, such as the 2020 pandemic or the climate crisis, only lead to moot structural changes in society. Without distancing ourselves from the transformation bias, we can’t even begin to ask the question about why tragic ruptures (e.g., disasters) often don’t lead to any transformation.

Clearly separating imagined and actual ruptures enables us to examine, more precisely, the role of rupture narratives within actual ruptures. We have suggested that narratives can alert us to early warning signs, canalizing and potentially blocking thinking, but also encouraging us to think differently. Imagined ruptures are not mirror reflections of ruptures, rather, they are active elements within ruptures – only rarely having transformative effects.

The function of imagined ruptures is not only to help us navigate actual ruptures (Zittoun et al., 2003). They also answer to our desires (Freud, 1899, 1907) and exploit our cognitive tendencies (Tobin, 2018). Accordingly, rupture narratives are multi-functional, and, as such, they can sometimes be misleading within an actual rupture. Rupture narratives pander to our fears and flatter our heroism. They come from a past, yet, they are applied to the future, and thus, inherently limited. People, we suggest, are often slow to recognize a rupture, and fail to make it a transformative experience, because they are cruising along unquestioned cultural channels of thought and action.

In so far as ruptures indicate a failure of cultural guidance, a key question becomes: what cultural guidance is available when cultural guidance fails? Our analysis of rupture narratives points to protagonists’ frequently rejecting advice and going rogue. While this doubtless panders to our desires for heroism, it may also be a form of meta-guidance. Namely, it could be interpreted as a cultural reminder to ignore culture when necessary. This is not to say that we can ever step outside culture, but it might encourage us to take a leap of faith, and jump to another canalized cultural track.

## Data Availability

No datasets were generated or analysed during the current study.
